# Stage 1 Registered Report: The relationship between handedness and language ability in children

**DOI:** 10.12688/wellcomeopenres.15077.1

**Published:** 2019-02-14

**Authors:** Verena E. Pritchard, Stephanie A. Malone, Kelly Burgoyne, Michelle Heron-Delaney, Dorothy V.M. Bishop, Charles Hulme

**Affiliations:** 1 Institute for Learning Sciences & Teacher Education, Australian Catholic University, Brisbane, Australia; 2 Education, University of Oxford, Oxford, Oxfordshire, OX2 6PY, UK; 3 Human Communication, Development and Hearing (HCDH), University of Manchester, Manchester, UK; 4 Experimental Psychology, University of Oxford, Oxford, UK

**Keywords:** Laterality, Language, Developmental Language Disorder

## Abstract

Weak or inconsistent hand preference may be a risk factor for developmental language delay.  This study will test the extent to which variations in language skills are associated with the strength of hand preference. Data are drawn from a large sample (n = 569) of 6- to 7-year-old children unselected for ability, assessed at two time points, 6 months apart. Hand preference is assessed using the Quantitative Hand Preference task (QHP) and five uni-manual motor tasks. Language skills (expressive and receptive vocabulary, receptive grammar, and morphological awareness) are assessed with standardized measures. If weak cerebral lateralisation (as assessed by the QHP task) is a risk factor for language difficulties, it should be possible to detect such effects in the large representative sample of children examined here.

## Relationships between handedness and language skills in children

Humans typically show cerebral lateralization for language. For the majority of the population, language processing appears to depend predominantly on left hemisphere systems. Much of the early evidence for cerebral lateralisation came from studies of adult stroke patients which showed that damage to the left hemisphere is more commonly associated with language deficits than damage to the right hemisphere ( Benson & Ardila, 1996; Damasio, 1992; Geschwind, 1971; Kertesz & McCabe, 1977). Patterns of cerebral lateralisation for language are also associated with measures of hand preference/hand function such that most adults with left-hemisphere dominance for language also show greater dexterity with the right hand ( Khedr *et al*., 2002: Knecht *et al*., 2000; cf. Mazoyer *et al*., 2014). This has led to the use of handedness as a marker for the cerebral lateralisation of language. Evidence shows that left-handers are indeed more likely than right-handers to have atypical lateralisation for language ( Knecht *et al*., 2000; Szaflarski *et al*., 2002); around 30% of left-handers vs 5% of right-handers have atypical lateralization for speech.

Studies of cerebral lateralisation in adults lead naturally to ideas about possible links between the development of cerebral lateralisation and language skills in children. If the development of cerebral lateralisation is critical for the development of language, handedness (as a proxy for the cerebral lateralisation of language) might be expected to relate to developmental language difficulties ( Annett, 2002; Bishop, 2013; Bishop *et al.*, 2014). In line with this, in some studies right-handedness has been reported to be associated with better language and literacy skills (see Somers *et al*., 2015 for a review). However, evidence for such associations is mixed. In their meta-analysis which included studies of both children and adults, Somers *et al*. (2015) found no overall difference in verbal ability between right- and left-handed people (Hedges’ *g* = −0.03, *p* = 0.22). A follow up analysis of studies that included only children reported a very small effect favouring right-handed children (Hedges’ *g* = −0.09) though this effect was reduced to nonsignificant levels after excluding two studies with disproportionately large sample sizes (Hedges’ *g* = −0.06). It seems clear from the Somers *et al*. meta-analyses that any association between hand preference (treated as a binary variable; left vs. right) and language ability in the general population is trivial in size, irrespective of age.

The absence of any clear relationship between handedness and language skill in the Somers *et al*. (2015) meta-analysis might reflect the fact that manual laterality is at best a weak proxy for language lateralisation in the brain ( Groen *et al*., 2013). In response to this, some studies have used physiological measures of laterality (e.g., functional Transcranial Doppler Ultrasound). Some of these studies have found evidence for differences in language laterality in children with language difficulties (Developmental Language Disorder (DLD); see Wilson & Bishop, 2018 for a review). However, in the largest study of this sort Wilson and Bishop failed to replicate such an association and current evidence suggests that there is little, if any, relationship between physiological measures of language laterality and language skills.

There is, however, a more nuanced view of the possible relationship between laterality and language skill that needs to be considered. In general, individual differences in laterality are seen as stable characteristics, but there is also evidence that some aspects of laterality mature with age: children progress from a rather ambivalently expressed hand preference to a more consistent hand preference ( Scharoun & Bryden, 2014). In this view, the delayed development of cerebral lateralisation may be associated with language difficulties. This implies that children who show weak or inconsistent hand preference may be at risk for developmental language disorder (DLD). Note that on this view, left- versus right-handedness is not expected to be associated with language status: the crucial aspect is not the direction of laterality, but rather the consistency of that lateralisation, whether to left or right.

To assess this idea, Bishop and colleagues ( Bishop *et al*., 1996) developed a measure of Quantitative Hand Preference (QHP). In the QHP task the person stands in front of a table with a set of cards arranged on either side of the midline. The task is simply to pick up cards one at time (in response to a verbal command signifying the picture on the card) and place them in a box at the midline. This task gives a simple quantitative measure of hand preference (the proportion of cards picked up with the right hand) on a task that appears to have minimal cultural influence. Using the QHP task, Hill & Bishop (1998) reported that 7- to 11-year-old children with DLD and children with developmental coordination disorder showed less clearly defined hand preference on the QHP task than age-matched controls (but similar levels of hand preference to a younger control group who were roughly 3 years younger). Because performance on the QHP task was impaired in both children with language and motor disorders, Hill and Bishop concluded that the “QHP task appears to be a sensitive, but non-specific, indicator of developmental disorders” (p. 295). A note of caution is needed however, since the group sizes in the Hill and Bishop study were small (12 children with motor difficulties, 20 children with language difficulties and 26 age-matched controls). However, other studies by Bishop and colleagues that have used the QHP with larger samples (i.e., Bishop, 2001; Bishop, 2005; Hill & Bishop, 1998) do indicate that the QHP is generally more successful than traditional hand preference inventories such as the Edinburgh Handedness Inventory ( Oldfield, 1971) in differentiating between children with specific language impairment and age matched controls.

Although the Wilson & Bishop (2018) findings are not encouraging, a stronger test of a maturational hypothesis would involve testing children's consistency of lateralisation at two ages. We would predict that we should see a shift from less consistent to more consistent lateralisation over time, and that earlier establishment of consistent preference would be correlated with better language skills.

If weak cerebral lateralisation (as assessed by the QHP) is a risk factor for language difficulties, it should be possible to detect such effects in large representative samples of children. Within such samples, children with the weakest language skills should be expected to show evidence of less clearly defined hand preference on the QHP. In the current study we use observations of hand preference and the QHP measure with a large unselected sample of children seen on two occasions. We will perform analyses treating language as a continuously distributed trait, as well as examining whether measures of handedness differ between children with particularly poor language skills versus controls.

## Methods

### Ethical considerations

The Australian Catholic University Human Research Ethics Committee provided ethical approval (2015-269H). Informed written consent was sought from the principals of the schools involved for all children enrolled in their first year of school (Preparatory Year) in January 2016. An opt-out procedure was followed. Parent information leaflets and opt-out consent forms were distributed to the parents of enrolled children (via both electronic and written hard copy format for each participant). All children in each class participated in the study unless parents signed the opt-out consent form for their child before the study’s commencement date. 

### Participants

A total of 569 children from 11 schools in Brisbane, Australia participated as part of a larger longitudinal study ( Burgoyne *et al*., submitted). The sample size was determined largely by constraints on funding. We recruited the largest sample that we could given the staffing levels available. The sample size is very large for a longitudinal study of cognitive development using individually administered measures. The schools selected are essentially a convenience sample and consist of a sub-sample of the schools located in the greater Brisbane area who were approached with a request to participate. According to Government data on the socio-economic composition of the population in each school (the Index of Community Socio-Educational Advantage; ICSEA) eight of the participating schools serve a student population with an average level of educational advantage (ICSEA values between 997 and 1090 where the average range (1 *SD* of the mean) is 900 to 1100). The three remaining schools have higher ICSEA values (1112–1153) reflecting a student population with slightly above average levels of social advantage. Children were assessed at two time points: within the final half of Year 1 (t4 *n* = 496; mean age 81.23 months; range 71–99 months), and again approximately 6 months later during the first half of Year 2 (t5; *n* = 454; mean age 87.74 months; range 77–106 months).

### Measures and procedure

 As part of the larger longitudinal study ( Burgoyne *et al*., submitted), children completed a battery of scholastic, cognitive and motor measures at t4 and t5 including measures of language and hand preference. All measures were administered individually in the children’s schools by two of the authors (SM; VP) and four postgraduate research assistants.

### Handedness

**Figure 1.**
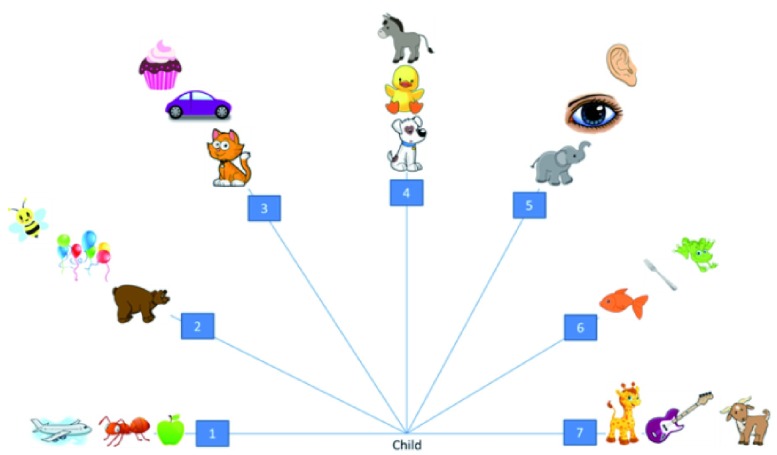
Illustration of the items and spatial positions in the Quantitative Hand Preference (QHP) task.

**Quantitative hand preference (QHP) task.** Bishop *et al*.’s (1996) QHP task was used to quantify the degree of hand preference. For this task children were required to stand and reach for individual picture cards one at a time placed on a waist-high table. The cards were positioned at one of seven positions extending at 30 degree intervals from the left to the right of the child’s midline to form a semi-circle. There are 21 trials in total (three cards spaced along each of the seven positions of which there are three to the left, one at midline, and three to the right). Children were asked to stand at the midline position and to pick up a named card and place it into a box directly in front of them. Figure 1 shows the items and set-up for the QHP task. Card selection followed a fixed random order and no time constraints were imposed. Reaching was scored following Bishop, 2005: one point is awarded for each reach done with the right hand, 0 points for bimanual usage or unclear preference, and -1 point for each reach done with the left hand.

**Unimanual hand preference motor tasks.** Measures of the hand used while children completed the following 5 motor tasks at both t4 and t5 were recorded. (1) *Lace Threading* (Movement Assessment Battery for Children – Second Edition (Movement ABC-2; Henderson *et al*., 2007). This task requires children to thread a string through eight holes on a board – a practice trial of four holes is followed by two test trials; (2) *The* Movement ABC-2 Drawing Trails subtest. This requires children to trace a pattern with a pen between two lines without lifting the pen from the page or cross the lines - one practice trial and two test trials were completed; 3) the *Apples Selection* task ( Breckenridge, 2008). This requires children to identify as many red apples as they can ( *n* = 30) printed on a page within a 60 second time limit while ignoring white distractor apples and red distractor strawberries; 4) the *Two-Match Shifting* task; Dick, 2014). In this task children are presented with a series of three boxes containing picture arrays. These pictures varied from each other along four dimensions: 1) object (boat, rose, rabbit); 2) colour (red, green, blue); 3) size (small, medium large); 4) quantity (one, two, three), and for each test trial children were asked to point to two pictures in one box that were the same as each other on one dimension but different from the pictures in the other box. For each test trial, there were two possible ways that the boxes could match - children completed 12 test trials; 5) *Token Placing* ( Cohen, 1997). In this task children are shown a 4 × 4 grid with a pattern of 8 red dots for 5 seconds and asked to recreate it on an empty grid using plastic discs – five test trials were completed for one pattern, and one trial for a different pattern.

### Language

**Expressive vocabulary**. The Expressive Vocabulary subtest from the CELF 4 ^AU^ ( Semel *et al*., 2006) was used. In this test, children are asked to name a series of pictures depicting objects (e.g. skeleton, saxophone) or actions (e.g. drawing). Testing was discontinued after 7 consecutive errors. Each response is scored as 0 (incorrect), 1 (partial response) or 2 (correct response). 

**Receptive vocabulary.** The Receptive One-Word Picture Vocabulary Test ( Brownell, 2010) was used to assess children’s’ *Receptive Vocabulary*. For this test, children were presented with four pictures and asked to point to the picture that matched the word spoken by the examiner. All children started at the item corresponding to the 7.0-7.11 age bracket. A basal level was established by scoring eight consecutive correct responses, and a ceiling was established by scoring six incorrect responses within eight consecutive items. Testing was discontinued after the ceiling had been established. A score of 1 was awarded for each correct response.

**Receptive grammar**. A shortened version of the Test for Reception of Grammar – Second Edition (TROG-2; Bishop, 2003) was used to assess children’s understanding of grammatical contrasts. The version of the TROG-2 used in the current study included 40 stimulus items arranged in blocks of 2, which test 20 grammatical contrasts (e.g. the prepositions “in” and “on”, pronouns, relative clauses). For each item, children were presented with a four-picture array (one target item and three distractor items including lexical and/or grammatical foils) and asked to point to the picture that best represents the grammatical or lexical element contained in the target sentence produced by the examiner.

**Morphological awareness.** Children’s morphological awareness was assessed with a Word Analogy task ( Kirby *et al*., 2012) that includes both inflectional and derivational transformations. In this task, children are/were asked to provide a missing word based on an analogical pattern for 10 inflectional items and 10 derivational items. For these, the experimenter would say a pair of words, for which the second word included a morphological shift. Then a target word was spoken and children were asked to apply the same morphological shift to this word as in the first pair (e.g., walker:walk::teacher: *teach* for inflection, and sleep:sleepy::cloud:: *cloudy* for derivation). A series of six practice items were provided first in which children were corrected if they gave an incorrect response. The child’s score was the total number of correct answers for both inflected and derived words.

## Analysis plan

One complication in these data is that children can be either right or left handed. It can be expected that roughly 90% of the sample will be right-handed. The analyses we propose will initially be conducted on right- and left-handed children separately. We will proceed to combine these samples if initial analyses of the separate groups support this. We have small amounts of missing data at each time point, and the data at t5 is for a slightly reduced sample compared to t4, missing data will be handled by pairwise deletion.

We will define handedness by the hand used in the Drawing Trails task.

We will measure performance on the QHP by the proportion of reaches made with the preferred hand.

We will address a series of questions by performing the following analyses:

### Primary research questions – The reliability of the QHP measure and the relationship between hand preference on the QHP and language ability

1. How reliable is the QHP measure of hand preference? We will assess the test-retest reliability of the QHP. This is the correlation between the proportion of reaches with the preferred hand at time 4 and time 5. We will compute three correlations: 1. For the right-handed sample; 2. For the left-handed sample; 3. For the combined sample.
2. Do children with language difficulties show weaker hand preference on the QHP than children without language difficulties? We will compute a language factor score based on the 4 measures of language ability that are available at both time 4 and time 5 (CELF Expressive Vocabulary; the Receptive One-word Picture Vocabulary Test; the Test for Reception of Grammar; and the Word Analogy task). We will use independent sample t-tests to assess whether children with language difficulties (those with language factor scores less than or equal to 1 standard deviation below the mean) make a lower proportion of reaches with the preferred hand than children without language difficulties (the rest of the sample). We will initially perform these tests separately for the t4 and t5 data and separately for right vs. left handers (four separate independent samples t-tests). If however, the patterns for left and right handers look comparable we will combine them at each time point to give tests of greater statistical power.
3. In the population as a whole, do variations in language skills correlate with strength of hand preference on the QHP? We will assess whether there is a relationship between language factor scores (treated as a continuous variable) and the proportion of reaches with the preferred hand on the QHP. We will perform linear regression analyses 1. For right handers; 2. For left handers and (assuming the relationships seem similar for the two samples); 3. For the combined sample. In all of these regression models we will examine the adequacy of a linear model and check for any undue influence of outliers. If there is evidence of a non-linear relationship between hand preference and language scores we may use quantile regression to explore this further.

### Secondary research questions – Possible developmental effects on the QHP measure, and relationships between QHP performance and other measures of handedness

4. Does the strength of hand preference (proportion of reaches with the preferred hand) increase with age (is it higher at time 5 than time 4?). Any such increase could be taken as evidence of maturation or alternatively merely evidence of a practice effect. For children in the sample who were tested at both time points we will compute 3 paired-samples *t*-tests comparing the mean proportion of reaches with the preferred hand 1. For the right-handed sample, 2. For the left-handed sample; 3. For the combined sample
5. Is the strength of hand preference (the proportion of reaches with the preferred hand) equivalent in right and left handers (or are left handers less strongly lateralized?). We will compute 2 independent samples t-tests comparing the proportion of reaches on the QHP task with the preferred hand in right vs left handers at both times of measurement (time 4 and time 5).
6. Do QHP scores differ for right-handed children with a consistent versus inconsistent hand preference as found by Bishop *et al*. (1996)? Consistency of hand preference here will be defined by the 5 motor tasks described above; consistent hand use will be defined as a child who uses the same hand for all 5 tasks. We will compute 2 independent samples t-tests comparing the proportion of reaches with the preferred hand on the QHP in 1. consistent vs. inconsistent right handers and 2. consistent vs. inconsistent left handers (the sample size for left handers will be small so the power in this latter analysis may be low).

### Study timeline

The dataset is a secondary registration of a pre-existing dataset. Data was collected across two time points separated by a six month interval: Time 4 (t4) in the last half of children’s second school year (August – December, 2017); Time 5 (t5) in the first half of their third school year (February – July, 2018). Assuming that Stage 1 review is successful we would envisage completing the report of this study within 6 months of the Stage 1 reviews being completed.

### Self-certification statement

The protocol proposed here is a secondary analysis of an existing dataset. CH, VP, SM, MH, and KB have had prior access to the data that will be used as part of this study. No dissemination of the dataset or of any works relating to the dataset has preceded this analysis plan.

The statistical analysis plan proposed was developed in collaboration with DB. DB was blinded to the data during the development of the analysis plan.

## Data availability

*No data is associated with this article*
