# Supplementary material for: Stage 1 Registered Report: The relationship between handedness and language ability in children
Source: Wellcome Open Res. 2019 Feb 14;4:30. [Version 1] doi: 10.12688/wellcomeopenres.15077.1 (PMC6415320; doi:10.12688/wellcomeopenres.15077.1)
Supplement: Peer review history

# Peer review: Reviewer response for version 1

**DOI:** 10.21956/wellcomeopenres.16449.r34873

**Published:** 2019-03-12

**Authors:** Todd Kettler

**Affiliations:** 1 School of Education, Baylor University, Waco, TX, USA

I answered “partly” to the item about the dataset being presented in a usable and accessible format. There was no dataset available. However, the authors sufficiently described the data. There was indication that there was some missing data that would be handled with pairwise deletion. While I understand the appeal of pairwise deletion to maintain as many participants as possible, there may be some question of how the pairwise deletion verified the missing at random nature of the missing data. Ultimately this is hard to assess on review when I do not know the amount of the missing data. I encourage authors to carefully and thoroughly address the amount and nature of the missing data to support their use of pairwise deletion.

 I believe the authors have pre-specified sufficient outcome-neutral tests to test the stated hypotheses. In some cases the power will be reduced because of the small sample size in left handers, but the authors seem to acknowledge this up-front. I encourage the authors to include a comprehensive correlation matrix so that the relationships between all variables are evidenced and could be useful on subsequent re-analyses.

**Have the authors pre-specified sufficient outcome-neutral tests for ensuring that the results obtained can test the stated hypotheses, including positive controls and quality checks?**

 Yes

I have read this submission. I believe that I have an appropriate level of expertise to confirm that it is of an acceptable scientific standard.

# Response

**Published:** 2019-03-14

**Authors:** Charles Hulme

**Affiliations:** Professor, UK

We thank the reviewer for these thoughtful responses.

We will ensure that we document the amounts of missing data thoroughly. 

We will check for whether missing data can be considered Missing Completely At Random. If this assumption appears to hold different approaches to dealing with missing data it should not introduce bias. We can also assess the robustness of our analyses by comparing the results of analyses based listwise and casewise deletion as well as by using Maximum Likelihood estimators to deal with missing values.

# Peer review: Reviewer response for version 1

**DOI:** 10.21956/wellcomeopenres.16449.r34867

**Published:** 2019-03-12

**Authors:** Torsten Baldeweg

**Affiliations:** 1 Developmental Neurosciences, UCL Great Ormond Street Institute of Child Health, University College London, London, UK

The proposed tests are appropriate. I would urge the authors to consider the contribution of children who might be scoring in the ‘clinical’ range for QHP and the unimanual tasks – given the known comorbidity of DLD and DCD ^1^^,^^2^. It could be important to exclude them, to ensure that the associations are not driven by low scoring individuals.

 Also, if available, a measure of general/fluid cognitive ability (matrices) or processing speed could be included as covariate to see if the associations are of a specific cognitive nature or related to domain-general abilities.

**Have the authors pre-specified sufficient outcome-neutral tests for ensuring that the results** **obtained can test the stated hypotheses, including positive controls and quality checks?**

 Yes

I have read this submission. I believe that I have an appropriate level of expertise to confirm that it is of an acceptable scientific standard.

## References

[1] HillEL: Non-specific nature of specific language impairment: a review of the literature with regard to concomitant motor impairments. *Int J Lang Commun Disord*.36(2) :149-71 1134459210.1080/13682820010019874

[2] VisscherCHouwenSMoolenaarBLyonsJScherderEJHartmanE: Motor proficiency of 6- to 9-year-old children with speech and language problems. *Dev Med Child Neurol*.2010;52(11) : 10.1111/j.1469-8749.2010.03774.x e254-8 10.1111/j.1469-8749.2010.03774.x 20804514

# Response

**Published:** 2019-03-14

**Authors:** Charles Hulme

**Affiliations:** Professor, UK

We thank the reviewer for these thoughtful responses. 

Rather than exclude children with low scores we would prefer to examine scatter plots of non-linear relationships between QHP scores and language ability scores. There is no clear criterion for what would constitute being in the “clinical range” on the QHP.

We would prefer to not use additional covariates such as general ability or processing speed because there is no clear theoretical rationale for doing so and there are some conceptual and statistical objections to such an approach (Dennis, et al., 2009)

DENNIS, M., FRANCIS, D., CIRINO, P., SCHACHAR, R., BARNES, M., & FLETCHER, J. (2009). Why IQ is not a covariate in cognitive studies of neurodevelopmental disorders. *Journal of the International Neuropsychological Society,* *15*(3), 331-343. doi:10.1017/S1355617709090481
